# Executive Functions Do Not Mediate Prospective Relations between Indices of Physical Activity and Academic Performance: The Active Smarter Kids (ASK) Study

**DOI:** 10.3389/fpsyg.2017.01088

**Published:** 2017-06-29

**Authors:** Katrine N. Aadland, Yngvar Ommundsen, Eivind Aadland, Kolbjørn S. Brønnick, Arne Lervåg, Geir K. Resaland, Vegard F. Moe

**Affiliations:** ^1^Faculty of Teacher Education and Sport, Western Norway University of Applied SciencesBergen, Norway; ^2^Department of Coaching and Psychology, Norwegian School of Sport SciencesOslo, Norway; ^3^Network for Medical Sciences, University of StavangerStavanger, Norway; ^4^TIPS-Centre for Clinical Research in Psychosis, Stavanger University HospitalStavanger, Norway; ^5^Department of Education, Faculty of Educational Sciences, University of OsloOslo, Norway

**Keywords:** objectively measured physical activity, aerobic fitness, motor skills, structural equation modeling, elementary school, cognition

## Abstract

Changes in cognitive function induced by physical activity have been proposed as a mechanism for the link between physical activity and academic performance. The aim of this study was to investigate if executive function mediated the prospective relations between indices of physical activity and academic performance in a sample of 10-year-old Norwegian children. The study included 1,129 children participating in the Active Smarter Kids (ASK) trial, followed over 7 months. Structural equation modeling (SEM) with a latent variable of executive function (measuring inhibition, working memory, and cognitive flexibility) was used in the analyses. Predictors were objectively measured physical activity, time spent sedentary, aerobic fitness, and motor skills. Outcomes were performance on national tests of numeracy, reading, and English (as a second language). Generally, indices of physical activity did not predict executive function and academic performance. A modest mediation effect of executive function was observed for the relation between motor skills and academic performance.

**Trial registration:** Clinicaltrials.gov registry, trial registration number: NCT02132494.

## Introduction

There is a growing body of evidence to suggest that children derive cognitive benefits from participating in physical activity, with changes in cognitive function induced by physical activity proposed as a mechanism for improved academic performance (Howie and Pate, 2012; Tomporowski et al., 2015; Donnelly et al., 2016). For example, Howie and Pate (2012) hypothesized a model for the causal links in their systematic review, where cognitive function acts as a mediator in the relation between physical activity (as well as physical fitness, and sports participation) and academic performance. Across the range of cognitive functions it is the higher-level executive functions that are shown to benefit the most from physical activity (Hillman et al., 2009).

Executive functions encompass inhibition, working memory, and cognitive flexibility, functions that are distinguishable, but moderately correlated with each other (Miyake et al., 2000). Despite a rapid growth in studies investigating relations between physical activity and executive function and/or academic performance, most evidence is cross-sectional and only investigates single links. To our knowledge, only the studies by Rigoli et al. (2012) and Roebers et al. (2014) have tested the mediation effects of executive function. Rigoli et al. (2012) showed that working memory mediated the relation between motor coordination and academic performance in an adolescent sample. A major limitation in their study, however, was the cross-sectional design. In order to claim mediation, evidence of change would need to be demonstrated (Little, 2013). Roebers et al. (2014) examined the predictive value of fine motor skills, intelligence, and executive function on academic performance 2 years later in preschool children. They showed that executive function plays a role in the link between motor skills and academic performance, as the prediction of academic performance from fine motor skills and intelligence was no longer significant when executive function was added in the model. Furthermore, no significant effect was evident for a link between fine motor skills and executive function. Fine motor skills, intelligence, and executive function covaried, suggesting that executive function processes are shared mechanisms involved in both fine motor tasks and intelligence tests.

**Figure 1:**
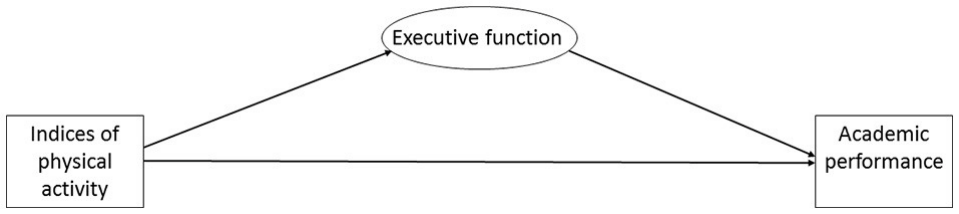
The hypothesized model.

Concerning this limited evidence for the executive function hypothesis, this study examined whether executive function mediated a possible prospective relation between indices of physical activity and academic performance in a cohort of 1,129 Norwegian elementary schoolchildren. The term “indices of physical activity” includes accelerometer measures [overall physical activity (counts per minute, cpm), moderate-to-vigorous physical activity (MVPA), and sedentary time], aerobic fitness, and motor skills. Our hypothesized model is illustrated in Figure 1.

The evidence for a direct link between physical activity and academic performance is mixed. A position paper published in 2016 concluded that physical activity has a neutral to positive effect on academic performance (Donnelly et al., 2016). The evidence base for this conclusion, however, is sparse and has important limitations, as randomized controlled studies of high quality are lacking (Singh et al., 2012; Mura et al., 2015; Donnelly et al., 2016). A recent intervention study by Mullender-Wijnsma et al. (2016) found improved performance in mathematics and spelling after 2 years of physically active mathematics and language lessons. However, neither the 5-month Learning, Cognition, and motion (LCoMotion) trial (Tarp et al., 2016), nor the 7-month ASK trial (Resaland et al., 2016), found evidence for an effect of physical activity interventions on academic performance.

There is stronger evidence for a positive relation between physical activity and executive function, than between physical activity and academic performance (Donnelly et al., 2016; Vazou et al., 2016). Laboratory research has reported both superior brain function and structure in more fit compared to less fit children (see Chaddock et al., 2011; Khan and Hillman, 2014 for reviews). However, the majority of these studies are cross-sectional and are thus unable to demonstrate a causal link.

Three pathways have been suggested by which physical activity could affect executive function (Best, 2010). First, participation in aerobic physical activity may induce physiological changes in the brain. Second, the cognitive demands inherent in goal-directed physical activities (e.g., group games) may also develop cognitive skills that can transfer to executive function tasks, and finally, the cognitive demands in executing complex motor tasks may induce physiological changes in the brain (Best, 2010). Hence, research has focused on both the quantitative (dose) and qualitative (type) characteristics of physical activity. These studies have reported both a dose-response relation between physical activity and executive functions (Davis et al., 2011; Hillman et al., 2014), and evidence for the importance of the cognitive demands inherent in physical activity through social interaction and complex motor skill tasks on executive functions (Crova et al., 2013; Schmidt et al., 2015; Pesce et al., 2016). Recently, Schmidt et al. (2015) demonstrated that although both an intervention with group-games and an intervention with individual aerobic exercise increased aerobic fitness, only the group-game intervention improved cognitive flexibility. Moreover, Pesce et al. (2016) showed the effects of an enriched physical education intervention consisting of both cognitively challenging activities and motor coordination compared to traditional physical education on inhibition. Despite the promising evidence of several pathways by which physical activity may affect executive functions, the effects of different kinds of physical activities (aerobic, coordinative, and cognitively engaging) on executive function is still unknown due to the large heterogeneity in the existing intervention studies investigating these questions (Vazou et al., 2016). The present study used objectively measured physical activity levels, aerobic fitness, and motor skills as predictors in separate mediation models, to investigate their possible different relations to executive function.

There is a clear link between executive functioning and academic performance (Bull and Scerif, 2001; St Clair-Thompson and Gathercole, 2006; Best et al., 2011; Bull and Lee, 2014; Cantin et al., 2016). Mathematics and reading are complex skills that reflect executive function skills such as selecting and coordinating different executive function components (Best et al., 2011). Even though the content in mathematics and reading are very different, their patterns of correlations to executive functions across age are similar (Best et al., 2011). This similarity indicates that the same cognitive processes are important to both reading and mathematics. The present study examines the relation between executive function and performance on national tests of numeracy, reading, and English (as a second language).

Against this evidence for the hypothesized links of indices of physical activity to executive function and academic performance, the present study extends previous research aiming to investigate whether executive function is a mediator in a prospective relation between indices of physical activity (objectively measured physical activity, aerobic fitness, and motor skill) and academic performance in numeracy, reading, and English. Since MVPA and sedentary time are separate dimensions of activity (Sedentary Behaviour Research Network, 2012), their possible different predictions of executive function and academic performance were examined. Furthermore, as sex-specific associations were observed between aerobic fitness and motor skills, and executive function and academic performance, in a previous study (Aadland et al., 2017a), we also examined if the mediation of executive function was different in girls and boys.

## Methods

The present study used data from the ASK study—a cluster-randomized controlled trial conducted in the county of Sogn og Fjordane, Norway, between August 2014 and June 2015. Sixty schools, encompassing 1,202 fifth-grade children, fulfilled the inclusion criterion of at least seven fifth-grade children enrolled, and agreed to participate. In total, 1,145 (82.1% of the population of 10-year-olds in the county) of the 1,175 invited children from 57 school agreed to participate. Valid data were provided from 1,129 children (Supplemental Figure 1). As there were no differences in physical activity levels (Resaland et al., 2016), aerobic fitness, and motor skills (Aadland et al., in press) between children in the intervention- and control group during the trial, both groups were included in the present study. We only provide a brief overview of relevant methods below as a detailed description of the study is given elsewhere (Resaland et al., 2015).

### Assessments

All assessments were conducted during school hours (between 08:30 a.m. and 2:30 p.m.), unless otherwise stated.

#### Physical activity and sedentary time

Physical activity and sedentary time were measured by ActiGraph accelerometers (ActiGraph GT3X+, LLC, Pensacola, Florida, USA), which is being widely applied and extensively tested for validity and reliability in children and youth (De Vries et al., 2009). Children were instructed to wear the accelerometer on the right hip at all time over seven consecutive days, except during water-based activities or while sleeping. A wear-time of ≥480 min/day for ≥4 days was applied as a criterion for a valid measurement. Periods of ≥20 min of zero counts were defined as non-wear time (Esliger et al., 2005). The outcomes for physical activity were overall physical activity (counts per minute, cpm), percent all day in MVPA (cut-point 2,296 cpm), and percent all day sedentary (0–100 cpm; Evenson et al., 2008; Trost et al., 2011). Files were analyzed at 10-s epochs using the KineSoft analytical software version 3.3.80 (KineSoft, Loughborough, UK).

#### Aerobic fitness

Aerobic fitness was measured with an intermittent practical running field test (the Andersen-test; Andersen et al., 2008; Aadland et al., 2014). The Andersen-test was administered according to standard procedures: Children ran from one end line to another (20 m apart) in an intermittent to-and-fro movement, with 15-s work periods and 15-s breaks (standing still), for a total duration of 10 min. Children were tested indoors on a wooden or rubber floor in groups of 10–20 children. We recorded the distance covered as the outcome for the analysis. To enable comparing of aerobic fitness level across studies, VO_2peak_ was calculated using the equation suggested by Aadland et al. (2014).

#### Motor skills

Motor skills were measured using a battery of three tests: (1) Catching with One Hand (Catching), (2) Throwing at a Wall Target (Aiming), and (3) Shuttle Run, 10 × 5 m. Tests 1 and 2 constitute the subgroup Aiming and Catching from the Movement Assessment Battery for Children 2 (Movement ABC-2), ageband 3 (11–16 years; Henderson et al., 2007), and test 3 is from the Eurofit test battery (Council of Europe, 1993). In accordance with the standard testing procedure for the Movement ABC-2, children performed five practice attempts in each task (1 and 2) before testing. No practice was given prior to the Shuttle Run test (3).

#### Executive functions

We measured key executive functions identified by Miyake et al. (2000); inhibition, working memory, and cognitive flexibility, by using four pen and paper tests. We assessed inhibition with the Stroop Color and Word Test (Golden, 1978). To assess cognitive flexibility, we used a semantic Verbal Fluency test (Spreen and Strauss, 1998), and The Trail Making Test (Spreen and Strauss, 1998; Lezak et al., 2012). Finally, we used a digit span test (digits forward and backward) from the Wechsler Intelligence Scale for Children, fourth edition (WISC-IV) to assess working memory (Lezak et al., 2012). All tests of executive functions are validated for use in children, and have been shown to be appropriate for measuring executive functions in 10-year-old children [Stroop (Peru et al., 2006), Verbal Fluency (Riva et al., 2000; Ardila et al., 2006), WISC-IV (Wechsler, 2003), and the Trail Making Test (Reitan and Wolfson, 2004)].

Trained research assistants tested the children individually in a quiet room at the child's school. All research assistants followed the same training and test procedures. On average, the test battery was completed in 15–20 min. For a more thorough description of the executive function tests, see Aadland et al. (2017a).

Although, the three-factor model of executive functions identified in young adults by Miyake et al. (2000) has also been confirmed in children (Lehto et al., 2003), age related differences in model solutions have been demonstrated (Brocki and Bohlin, 2004; Huizinga et al., 2006; St Clair-Thompson and Gathercole, 2006; Lee et al., 2013). This may indicate that executive function become more differentiated during childhood (Best and Miller, 2010). From the apparent interrelation of the executive functions in childhood, and the known impurity problems in executive function tasks (Best and Miller, 2010; Cassidy, 2016) we treated executive function as a single latent factor. Furthermore, a latent factor has increased reliability, as measurement errors are excluded (Cole and Maxwell, 2003). Variables included in the latent factor were the Color-Word task of the Stroop Test (Stroop CW), Verbal Fluency total, the Digit Backward of WISC-IV (WISC-IV backward), and the Trail Making Test part B (TMT-b).

#### Academic performance

Academic performance in numeracy, reading, and English was measured using specific standardized Norwegian National tests designed and administered by the Norwegian Directorate for Education and Training (NDET). The three different tests were administered on three different days. The tests have shown evidence of good validity and reliability by NDET and are aligned with the competencies demanded from all schools by the national curriculum (Resaland et al., 2015). The scores are reported as standardized points, with a mean of 50 and a standard deviation (SD) of 10.

### Potential covariates

Several covariates were controlled for in the analyses as they have been shown to affect the dependent and independent variables in the present study; age (Best and Miller, 2010; Kolle et al., 2010; Esteban-Cornejo et al., 2015), sex (Aadland et al., 2017a), body fat (Kolle et al., 2010; Davis and Cooper, 2011), pubertal status (Kalkut et al., 2009), and socio economic status (London and Castrechini, 2011). Body fat was measured using four skinfold thickness sites (biceps, triceps, subscapular, and suprailiac) using a Harpenden skinfold caliper (Bull; British Indicators Ltd., West Sussex, England) according to the criteria described by Lohman et al. (1991). The Harpenden skinfold caliper has been tested for validity and reliability in children (Yeung and Hui, 2010). Children self-assessed their pubertal stage with the Tanner method (Tanner, 1962) using a scale of colored images proposed by Carel and Leger (2008). We used breast and genital development for girls and boys, respectively. Socio economic status (the highest education level obtained by the mother and father) was reported by the parents or guardians. Furthermore, as we merged children from an intervention- and a control group into one cohort, we also controlled for group allocation in our mediation analyses. A more detailed description of the methods is provided in the design paper (Resaland et al., 2015).

### Ethics statement

The procedures and methods used in the ASK study conform to the ethical guidelines defined by the World Medical Association's Declaration of Helsinki and its subsequent revisions (WMA, 2013). The study protocol was approved by the Regional Committee for Medical and Health Research Ethics South East (REC South East). We obtained written consent from a parent or guardian of each child prior to all testing.

### Statistical methods

All study variables were examined for distributional properties. TMT-b was transformed (1/x) while other variables were left in their original form. We excluded all values exceeding five standard deviations from the mean. Children's characteristics are provided as means and standard deviations (SD), or frequencies.

A linear mixed model including school as a random effect was used to examine differences between sexes. A chi-square test was used to test for differences between sexes in pubertal status and socio economic status. The descriptive analyses were conducted with SPSS software, version 23.0 (IBM SPSS Statistics for Windows, Armonk, NY: IBM Corp., USA).

Structural equation modeling (SEM), with full information maximum likelihood estimation (FIML) was used to examine the mediation models and the bivariate correlations. The analyses were implemented through Mplus, version 7.4 (Muthén and Muthén, Los Angeles, USA). Because we only had two time points of measurement, we used a half-longitudinal mediation approach as explained in Cole and Maxwell (2003) and Little (2013). As it is possible for a predictor to have an indirect effect on academic performance through executive function, without a direct effect between the two, we tested the full mediation models and did not use the causal steps approach by Baron and Kenny (1986) and Hayes (2009). We used seven predictor variables (cpm, MVPA, sedentary time, aerobic fitness, Shuttle Run, Aiming, and Catching) and three outcome variables (numeracy, reading, and English), resulting in 21 different mediation models. Each mediation model was conducted in two steps, advancing in complexity; (1) mediation models including covariates, by adding a regression from each covariate to all dependent and independent variables, and (2) mediation models examining sex-differences, by conducting a multi-group analysis (covariates included). As it is not possible to take into account the cluster effect while using the bootstrap command, each model was furthermore performed twice; once including cluster (MLR estimator) and once with bias corrected bootstrap (10,000 bootstrap samples). We used bootstrapping to construct asymmetric confidence interval for the indirect effects (ab). With bootstrapping, the confidence intervals are based on an empirical generated representation of the sampling distribution of ab that respect the fact that indirect effects can be extremely non-normally distributed.

Due to the large sample size, multiple indices in addition to the chi-square test statistic were used to assess model fit; the Comparative Fit Index (CFI), the Root Mean Squared Error of Approximation (RMSEA), and the Standardized Root Mean Square Residual (SRMR). We used a non-significant χ^2^ and the cutoff recommendations of CFI > 0.95, and RMSEA and SRMR < 0.05 as indications of good model fit to the data (Geiser, 2013).

Measurement invariance was tested for the latent factor of executive function both across time and across sex. A *p* ≤ 0.05 was used to indicate statistical significance in all analyses.

## Results

The children's characteristics are shown in Table 1. Girls performed better on all but one test of executive function, while boys did better on the tests of numeracy and English. Boys had higher physical activity levels, better aerobic fitness, and better motor skills than girls, whereas girls had higher skinfold thickness and more advanced pubertal status than boys. A correlation matrix for the included independent and dependent variables is provided in Table 2.

**Table 1:** Baseline characteristics of the children as means and standard deviations (SD) or frequencies.

	**Girls**		**Boys**		**Total**	
**Variable**	***n***	***M (SD)/%***	***n***	***M (SD)/%***	***n***	***M (SD)/%***
Age (years)	541	10.2 (0.3)	588	10.2 (0.3)	1,129	10.2 (0.3)
BMI	531	18.1 (3.0)	564	18.0 (3.0)	1,095	18.1 (3.0)
Body fat (mm)	527	58.4 (29.3)	557	42.2 (20.9)^***^	1,084	50.1 (26.6)
Pubertal stage (Tanner) (%)	526		555		1,081	
Stage 1	116	21.4	193	32.8^***^	309	27.4
Stage 2	345	63.8	303	51.5	648	57.4
Stage 3, 4, and 5	65	12.2	59	10.0	124	11.0
Socio economic status (%)	511		558		1,049	
≤ Upper secondary school	156	28.8	193	32.8	349	30.9
<4 years of university/college	156	28.8	164	27.9	320	28.3
≥4 years of university/college	199	36.8	201	34.2	400	35.4
PA-levels (full day)						
Counts per minute (cpm)	484	691 (236)	521	773 (299)^***^	1,005	733 (274)
SED (% all day)	484	60.2 (5.9)	522	59.5 (6.5)	1,006	59.8 (6.2)
MVPA (% all day)	484	8.9 (2.7)	522	10.5 (3.5)^***^	1,006	9.7 (3.3)
Aerobic fitness (m)	511	868.6 (85.8)	534	915.9 (112.6)^***^	1,045	893.8 (103.1)
Estimated VO_2peak_ (ml/kg/min)	510	48.9 (6.9)	534	55.2 (7.3)	1,044	52.3 (8.0)
Motor skills						
Shuttle Run (s)	527	23.6 (2.2)	556	22.7 (2.3)^***^	1,083	23.1 (2.5)
Aiming (n)	532	3.8 (1.9)	561	4.2 (1.9)^***^	1,093	4.0 (1.9)
Catching (n)	507	3.3 (2.9)	526	4.8 (3.1)^***^	1,033	4.1 (3.1)
Executive function						
Stroop CW (n)	525	26.6 (5.8)	563	25.1 (6.0)^***^	1,088	25.8 (5.9)
Verbal Fluency (n)	528	16.0 (4.6)	567	16.0 (4.7)	1,095	16.0 (4.6)
WISC-IV backward (n)	526	6.3 (1.4)	567	6.1 (1.3)^**^	1,093	6.2 (1.3)
TMT-b (s)	512	114.9 (40.8)	529	128.6 (53.3)^***^	1,051	121.9 (48.1)
Academic performance						
Numeracy	518	50.3 (8.9)	562	52.1 (9.9)^***^	1,080	51.3 (9.5)
Reading	513	49.7 (9.4)	553	49.2 (10.0)	1,066	49.4 (9.7)
English	515	48.6 (9.0)	547	50.1 (10.5)^*^	1,062	49.4 (9.8)

*BMI, body mass index; PA, physical activity; SED, sedentary time; MVPA, moderate-to-vigorous physical activity; Stroop CW, Stroop Color Word; WISC-IV, Wechsler Intelligence Scale for Children fourth edition; TMT-b, Trail Making Test part B*.

* p ≤ 0.05 for the difference between girls and boys;

** p ≤ 0.010 for the difference between girls and boys;

*** *p ≤ 0.001 for the difference between girls and boys*.

**Table 2:** Estimated correlation matrix for the independent and dependent variables at baseline (above the diagonal line) and at follow-up (below the diagonal line).

	**1**	**2**	**3**	**4**	**5**	**6**	**7**	**8**	**9**	**10**	**11**	**12**	**13**	**14**
1.cpm	—	**0.88**	−**0.67**	**0.23**	−**0.20**	**0.10**	**0.25**	−0.01	0.05	0.01	0.02	0.10	−0.01	0.00
2.MVPA	**0.90**	—	−**0.75**	**0.35**	−**0.27**	−**0.11**	**0.29**	−0.00	0.04	0.02	0.01	**0.12**	0.00	−0.00
3.SED	−**0.68**	−**0.72**	—	−**0.18**	**0.13**	−0.05	−**0.14**	0.04	−0.05	0.04	0.01	−0.05	0.04	**0.11**
4.Aerobic fitness	**0.37**	**0.47**	−**0.25**	—	−**0.58**	**0.23**	**0.38**	**0.17**	**0.09**	**0.14**	**0.16**	**0.23**	**0.10**	**0.08**
5.Shuttle Run	−**0.23**	−**0.30**	**0.17**	−**0.66**	—	−**0.24**	−**0.39**	−**0.20**	−**0.13**	−**0.13**	−**0.16**	−**0.28**	−**0.19**	−**0.16**
6.Aiming	**0.17**	**0.22**	−**0.14**	**0.31**	−**0.29**	—	**0.36**	**0.15**	**0.09**	**0.07**	**0.10**	**0.13**	0.05	0.04
7.Catching	**0.25**	**0.31**	−**0.14**	**0.42**	−**0.41**	**0.36**	—	**0.18**	**0.08**	**0.12**	**0.17**	**0.19**	**0.10**	**0.09**
8.Stroop CW	−0.05	−0.03	**0.08**	**0.17**	−**0.19**	**0.16**	**0.15**	—	**0.14**	**0.28**	**0.41**	**0.33**	**0.32**	**0.31**
9.Verbal Fluency	−0.04	−0.02	0.01	**0.07**	−**0.09**	0.05	−0.02	**0.18**	—	**0.17**	**0.23**	**0.24**	**0.25**	**0.19**
10. WISC−IV backward	−0.01	0.02	0.04	0.05	−**0.07**	**0.09**	**0.08**	**0.29**	**0.13**	—	**0.30**	**0.33**	**0.32**	**0.30**
11.TMT−b	−02	0.02	−0.00	**0.12**	−**0.16**	**0.13**	**0.13**	**0.40**	**0.20**	**0.28**	—	**0.44**	**0.36**	**0.32**
12.Numeracy	0.05	**0.09**	0.04	**0.27**	−**0.31**	**0.18**	**0.20**	**0.38**	**0.20**	**0.34**	**0.42**	—	**0.67**	**0.60**
13.Reading	−0.02	−0.02	**0.11**	**0.17**	−**0.20**	**0.13**	**0.13**	**0.41**	**0.26**	**0.35**	**0.37**	**0.64**	—	**0.69**
14.English	−0.04	−0.03	**0.18**	**0.09**	−**0.11**	**0.05**	**0.06**	**0.32**	**0.17**	**0.28**	**0.25**	**0.51**	**0.64**	—

*Significant regression coefficients are in boldface. Cpm, counts per minute; SED, sedentary time; MVPA, moderate-to-vigorous physical activity; Stroop CW, Stroop Color Word; WISC-IV backward, Wechsler Intelligence Scale for children backward; TMT-b, the Trail Making test part B. R*.

### The latent executive function factor

As the score on the Verbal Fluency test made a small contribution to the latent executive function factor (baseline: *R*^2^ = 0.095 vs. *R*^2^ = 0.206–0.481 for other variables; follow-up: *R*^2^ = 0.085 vs. *R*^2^ = 0.205–0.402 for other variables), it was excluded. The latent factor of executive function showed metric and partial scalar invariance over time, with ΔCFI, ΔRMSEA, and ΔSRMR below suggested criteria (Putnick and Bornstein, 2016), as well as a non-significant Sartorra-Bentler scaled Chi-square. Comparing the metric model against the configural model gave Δ χ^2^ (Δ*df* = 2) = 1.436, ΔCFI <0.001, ΔRMSEA <0.001, and ΔSRMR = 0.005, and comparing the partial scalar model against the metric model gave Δ χ^2^ (Δ*df* = 1) = 0.096, ΔCFI <0.001, ΔRMSEA <0.001, and ΔSRMR = 0.006. The intercept of the WISC-IV backward was varied over time.

### The mediation models

**Figure 2:**
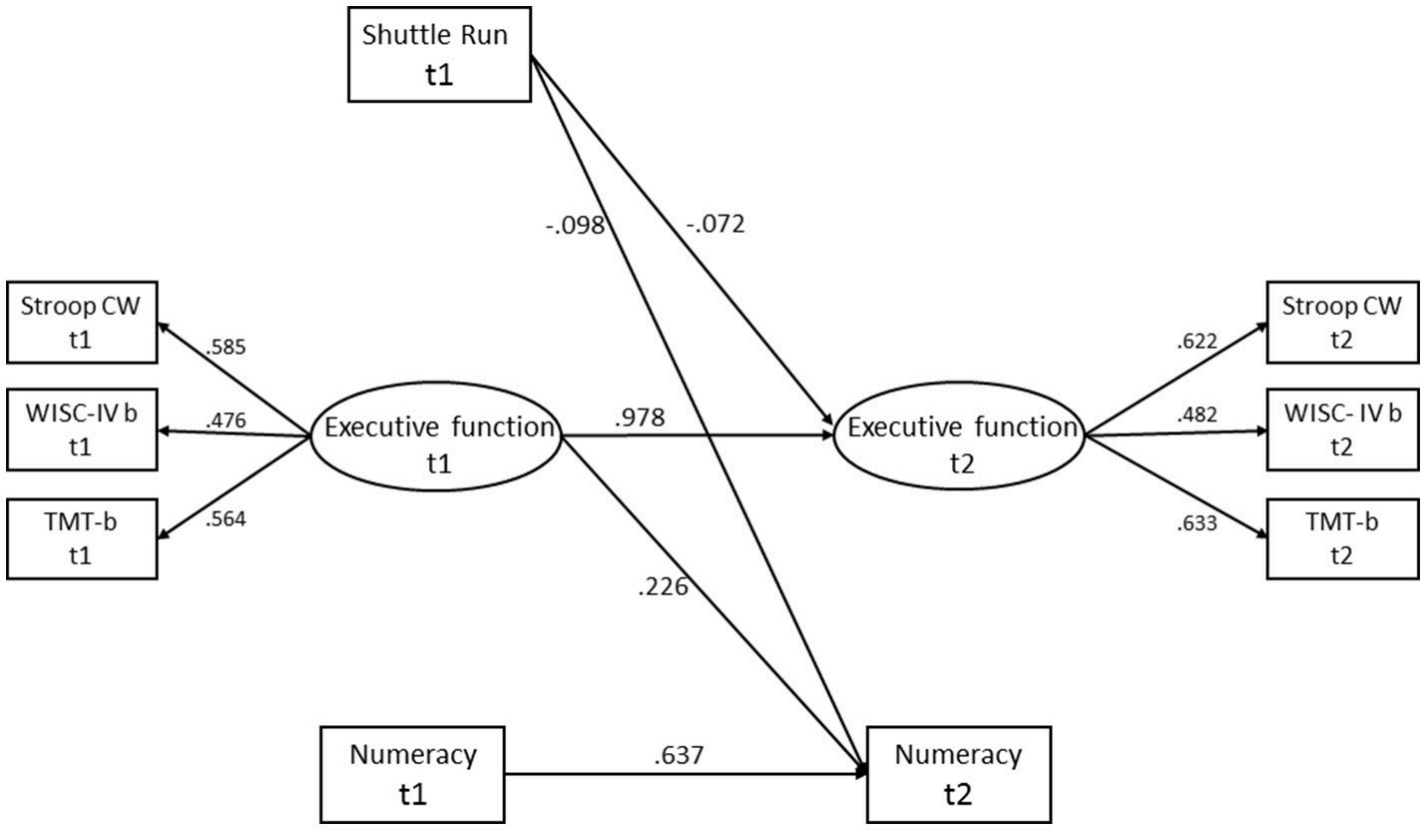
The half-longitudinal mediation model for executive function in the relation between Shuttle Run and numeracy. All path coefficients are significant and reported as standardized β-estimates. The covariates age, sex, tanner, body fat, socio economic status, and group allocation are adjusted for in the model, but not shown. t1, baseline; t2, follow-up; Stroop CW, Stroop Color Word; WISC-IV backward, Wechsler Intelligence Scale for children fourth edition backward; TMT-b, the Trail Making test part B.

As shown in Table 3, all models had good fit. When comparing each model adjusted for cluster against the same models with bootstrapping the results were nearly identical. Generally, the indices of physical activity did not predict either executive function or academic performance when controlling for covariates, hence no mediation effect of executive function was observed. Yet, executive function partially mediated the relation between the performance on the Shuttle Run test and numeracy (Figure 2). Both direct and indirect effects were statistically significant with small estimates. Direct paths from the Shuttle Run test to reading and from sedentary time to English were also observed. Additionally, executive function significantly predicted numeracy and reading.

**Table 3:** Standardized coefficients for the paths and goodness of fit indices for the half-longitudinal mediation models controlled for covariates.

**Model**	**a**	**b**	**axb**	**c′**	**χ^2^**	**CFI**	**RMSEA**	**SRMR**
	**β**	**β**	**β**	**β**	**(*df)***		**(95% CI)**	
**NUMERACY**								
cpm	−0.081	0.245^***^	−0.020	0.027	47.386 (45)	0.999	0.007 (0.000–0.022)	0.017
MVPA	0.003	0.244^***^	0.001	0.035	46.553 (45)	0.999	0.006 (0.000–0.022)	0.017
SED	−0.048	0.246^***^	−0.012	−0.022	48.433 (45)	0.999	0.009 (0.000–0.023)	0.017
Aerobic fitness	−0.023	0.241^***^	0.010	−0.006	41.430 (45)	0.998	0.012 (0.000-0.025)	0.017
Shuttle Run	−0.072^*^	0.226^***^	−0.016^*^	−0.098	57.946 (45)	0.996	0.017 (0.000–0.028)	0.017
Aiming	0.000	0.245^***^	0.000	−0.004	52.645 (45)	0.997	0.013 (0.000–0.026)	0.017
Catching	−0.033	0.233^***^	−0.008	0.026	47.712 (45)	0.999	0.008 (0.000–0.023)	0.017
**READING**								
cpm	−0.005	0.187^***^	0.001	−0.004	47.126 (45)	0.999	0.007 (0.000–0.022)	0.017
MVPA	0.002	0.187^***^	0.000	−0.004	46.451 (45)	0.999	0.006 (0.000–0.022)	0.017
SED	−0.050	0.187^***^	−0.009	0.018	48.513 (45)	0.999	0.009 (0.000–0.023)	0.017
Aerobic fitness	−0.026	0.166^***^	−0.004	0.055	49.091 (45)	0.999	0.009 (0.000–0.024)	0.017
Shuttle Run	−0.066	0.169^***^	−0.011	−0.069^*^	53.192 (45)	0.997	0.013 (0.000–0.026)	0.018
Aiming	−0.006	0.190^***^	−0.001	−0.012	50.791 (45)	0.998	0.011 (0.000–0.025)	0.017
Catching	−0.040	0.183^***^	−0.007	0.012	45.726 (45)	1.000	0.004 (0.000–0.024)	0.017
**ENGLISH**								
**cpm**	−0.005	0.046	−0.000	−0.006	48.111 (45)	0.999	0.008 (0.000–0.023)	0.017
MVPA	0.002	0.047	0.000	−0.009	47.456 (45)	0.999	0.007 (0.000–0.023)	0.017
SED	−0.049	0.047	−0.002	0.056^*^	49.008 (45)	0.999	0.009 (0.000–0.023)	0.017
Aerobic fitness	−0.028	0.056	−0.002	−0.030	49.396 (45)	0.999	0.010 (0.000–0.024)	0.017
Shuttle Run	−0.065	0.046	−0.003	−0.002	53.242 (45)	0.997	0.013 (0.000–0.026)	0.018
Aiming	−0.005	0.046	−0.000	0.000	51.329 (45)	0.998	0.012 (0.000–0.025)	0.017
Catching	−0.041	0.048	−0.002	−0.005	46.347 (45)	1.000	0.005 (0.000–0.022)	0.017

a, the path between the predictor and executive function at timepoint 2; b, the path between the executive function at baseline to the outcome at follow-up; axb, the indirect effect; c', the path between the predictor and the outcome; cpm, counts per minute; MVPA, moderate-to-vigorous physical activity; SED, sedentary time;

* p ≤ 0.05;

*** *p ≤ 0.001*.

Scalar invariance was found across sex for the latent factor of executive function when comparing the scalar model against the configural model (*p* = 0.409). None of the mediation models was statistically different for girls and boys.

## Discussion

The main finding from the present study was that executive function generally did not mediate the prospective relations between indices of physical activity and academic performance. Supplementing a direct link between motor skills and numeracy, we observed a small partial mediation effect through executive function. Executive function predicted numeracy and reading 7 months later.

Our findings generally do not support a hypothesized model in which executive function mediates the relations between indices of physical activity and academic performance (Howie and Pate, 2012; Tomporowski et al., 2015; Donnelly et al., 2016). Our predictors were associated with neither executive function nor academic performance, and our findings contrast with the conclusions drawn in recent systematic reviews, based on a small number of high-quality studies (Singh et al., 2012; Donnelly et al., 2016), that identified a positive relation between indices of physical activity, academic performance and even more so executive function. To our knowledge, only Roebers et al. (2014) have previously examined whether executive function mediates the relation between fine motor skills and academic performance in children using a longitudinal design. As the present study measured multiple predictors, it adds knowledge about the genuine prospective role of different indices of physical activity to executive function and academic performance.

Neither physical activity, sedentary time, nor aerobic fitness predicted executive function or academic performance 7 months later indicate a consistent pattern of findings. Aerobic fitness is frequently used as a proxy of physical activity, as it is partly determined by the physical strain induced by MVPA, and also due to its superior measurement precision compared to physical activity. It has been hypothesized that the effect of physical activity on executive function and academic performance operates through aerobic fitness (Tomporowski et al., 2011), as physical activity increasing aerobic fitness causes physiological changes in the brain that influence cognitive functioning. This hypothesis is partly supported by the cross-sectional study by Lambourne et al. (2013) in which aerobic fitness mediated the relation between physical activity and performance in mathematics, but not in reading and spelling. However, their indirect estimate was very low (0.003). Our longitudinal study does not provide support for this hypothesis, as no link was observed between aerobic fitness and executive function at follow-up. However, previous longitudinal studies have revealed positive relation between aerobic fitness and both executive function (Niederer et al., 2011; Chaddock et al., 2012) and academic performance (London and Castrechini, 2011; Wittberg et al., 2012; Bezold et al., 2014). In contrast to the present study, Booth et al. (2014) observed that the proportion of time spent in MVPA predicted performance in English and mathematics. Aggio et al. (2016) observed that higher levels of sedentary time were associated with improved cognitive performance 3 years later. In the present study, higher levels of sedentary time were associated with increased performance in English, but not in numeracy or reading. An explanation for this finding might be that children partly learn their English from screen-based, and thus sedentary, forms of entertainment (Cliff et al., 2016).

A considerable heterogeneity exists across the studies examining relations between indices of physical activity and cognitive outcomes, opening up for inconsistent findings in the literature. We cannot exclude the possibility that we failed adequately to identify expected links as a result of measurement error or methodological problems. Although, all assessments used in the present study have been shown to be appropriate and valid for the included age group (Council of Europe, 1993; Riva et al., 2000; Wechsler, 2003; Reitan and Wolfson, 2004; Ardila et al., 2006; Peru et al., 2006; Henderson et al., 2007; Andersen et al., 2008; De Vries et al., 2009; Utdanningsdirektoratet, 2013; Aadland et al., 2014), our null-findings might be a type 2 error as a result of measurement errors. Although, accelerometer-determined physical activity is more reliable than self-report, it does nevertheless have well-known limitations (Ekelund et al., 2007; Corder et al., 2008). For example, our measure of physical activity levels over 4–7 days might be an insufficient snapshot of a child's complex physical activity behavior (Ekelund et al., 2007), despite reliability (intra-class correlation) of accelerometry of ~0.70–0.80 for monitoring in children over 3–7 days (Aadland and Johannessen, 2015). As is well known, measurement error in predictors can lead to regression dilution bias, which underestimates the paths between the predictors and the mediator (Cole and Maxwell, 2003). Nonetheless, the lack of a prospective link between MVPA and executive function has also been shown by others (Booth et al., 2013; Aggio et al., 2016). Underestimation of the link between the mediator and the outcome may also be present in our models, as a result of measurement errors in the academic performance scores we used.

Our failure to identify links between predictors and executive function, in contrast with the existing literature (Donnelly et al., 2016), might also be explained by the use of different statistical approaches to examine these relations. To our knowledge, only the study by Roebers et al. (2014) have previously used structural equation modeling, treating executive function as a latent factor. Our rationale for using a latent factor was two-fold; first, to take into account the known impurity issues observed in executive function tasks (Cassidy, 2016) as we only had one measure of each domain, and second, to avoid measurement errors. The exclusion of measurement errors in our mediator enhances reliability, and avoids underestimation of both the a and b path and an overestimation of the direct link which is the case for observational mediator variables (Cole and Maxwell, 2003). Possibly, using latent variables of each dimension of executive function might have yielded other results, as randomized controlled trials have reported effects of indices of physical activity on only one aspect of executive functions and not the others (Schmidt et al., 2015; Pesce et al., 2016). However, such an approach would have required a more comprehensive test battery, which was not feasible for the present study. The selection of executive function tasks might also explain our null findings, as we did not include tasks measuring reaction time or accuracy, which would have allowed more fine-grained analysis. For example, Syvaoja et al. (2014) observed cross-sectional associations between objectively measured physical activity and both reaction time and rapid visual information processing, but not with other tests of executive function.

The longitudinal design of the present study differs from the majority of existing evidence stemming from cross-sectional studies. Indeed, a cross-sectional examination of our mediation models supports the executive function hypothesis proposed in previous studies (Howie and Pate, 2012; Rigoli et al., 2012; Tomporowski et al., 2015; Donnelly et al., 2016). More specifically, indirect effects through executive function were present for the cross-sectional relations between both aerobic fitness and all tests of motor skills and academic performance in numeracy, reading, and English (results not shown). Yet, cross-sectional studies lack a temporal relation between the exposure and outcome, and are unable to demonstrate causation. Thus, the half-longitudinal approach applied in the present study is a significant improvement compared to cross-sectional testing of mediation, as we were able to control for prior levels of the mediator and the outcome, and thus examining the influence of their change (Little, 2013). The use of only two measurement time points however, poses a limitation to the present study, as the path between the predictor and the mediator, and the path between the mediator and the outcome, are measured at the same time point. An assumption therefore is that these paths would have had time-ordered relations if more than two occasions of measurement were obtained (Little, 2013). Hence, studies replicating our analysis with more than two time points of measurement are warranted.

Another explanation for our null findings may be the short duration of our follow-up period. 7 months might have been an insufficient duration to cause a change in executive function and academic performance. Assuming that change in executive function will result from structural changes in the brain, sufficient time for the predictor to affect structural changes in the brain is necessary. In order to observe changes in academic performance, it is possible that a longer time is necessary. For example, the study by Mullender-Wijnsma et al. (2016) found effects on academic performance after 2 years but not after 1 year of physically active mathematics and language lessons. Other studies using shorter physical activity intervention length (5–7 months) have not revealed effects on academic performance or executive functions (Resaland et al., 2016; Tarp et al., 2016).

On the other side, a 7-month follow-up period also impose noise. As a prerequisite for our prospective examinations, our indices of physical activity measured at baseline represents the child's supposed physical activity level, aerobic fitness, and motor skills during the follow-up period. However, we cannot rule out the possibility of fluctuations from the baseline measures. Levels of physical activity may have been less stable over the follow-up period (Jones et al., 2013), compared to aerobic fitness and motor skills, as it represents a behavior and not a personal trait. Following this line of reasoning, our observed significant prediction for the Shuttle Run test on both executive function and academic performance, might be explained by the stability of this trait over time. Furthermore, Pesce and Ben-Soussan (2016) suggest that motor skills have a longer-lasting predictive value of cognitive efficiency compared to aerobic fitness. A prospective association for motor skills with executive function has also been observed in previous studies (Niederer et al., 2011; Roebers et al., 2014).

Following up on the prediction of motor skills to executive function and academic performance, executive function mediated the relation between the Shuttle Run and academic performance in numeracy. These findings may be explained by the close parallelism of development and interaction between neural substrates of motor coordination (the cerebellum) and executive function the (prefrontal cortex; Diamond, 2000; Koziol et al., 2012; Rigoli et al., 2012). Likewise, the concept of embodied cognition directly links movements to thought, where executive functions are seen as an extension of the motor control system (Koziol et al., 2012). The review by Best (2010) suggests that engaging in activities that are complex in terms of motor coordination may transfer executive function skills to other contexts. Nevertheless, the magnitude of both the direct and indirect links between motor skills and academic performance in the present study was small (standardized coefficient of −0.016), emphasizing the need for more research examining this relation. Furthermore, taken into account the large number of mediation models analyzed, which potentially may increase the type 1 error rate, it could be a chance finding that should be interpreted carefully (Ioannidis, 2005). The lack of mediation for the two other measures of motor skills, Aiming and Catching, may support this line of reasoning. However, we found a low pre-to-post correlation for Aiming (*r* = 0.27), indicating poor reliability. On the contrary, pre-to-posttest correlations for Catching and Shuttle Run were 0.66 and 0.70, respectively. In contrast to the present study's findings, both the study by Rigoli et al. (2012) (cross-sectional) and Pesce et al. (2016) (RCT) highlight that the subgroup Aiming and Catching from the Movement ABC is linked to executive function and academic achievement.

Finally, our contrasting findings to the present literature might be attributable to publication bias in the literature, as also considered by Howie and Pate (2012). It is possible that positive findings have been highlighted despite mixed findings, or that the variables reported were selected on the basis of positive findings (Ioannidis, 2005; Howland, 2011; Howie and Pate, 2012).

We found that executive function predicts numeracy and reading, which is in line with previous research (St Clair-Thompson and Gathercole, 2006; Best et al., 2011; Cantin et al., 2016; Samuels et al., 2016). A close link between numeracy and reading has been demonstrated previously (Bull et al., 2008; Best et al., 2011), and a study by Cantin et al. (2016) revealed that reading mediated the influence of executive function on mathematics. The Norwegian national tests of both numeracy and reading reflect integrated tasks across several subjects, and require both problem solving and metacognition, with high demands on executive functions. The test in English, however, focuses on grammar and vocabulary. It is possible that these tasks put less demand on executive functions, explaining the lack of relation between executive functions and English. Randomized controlled trials have revealed that the effect of indices of physical activity to cognition are selective to aspects of cognition that required extensive amounts of executive functions (Kamijo et al., 2011; Hillman et al., 2014).

### Strengths and limitations

The main strengths of the present study were the longitudinal design with inclusion of a large sample of 10-year-old children. We furthermore adjusted for the effect of clustering of observations within schools, as well as several covariates in our analyses. However, we cannot rule out that factors being important for the relations examined were not taken into consideration; for example, the child's motivation, other academic activities, quality of life, home environment, nutritional habits, or sleep (Tomporowski et al., 2011). Another, strength is the use of structural equation modeling including a latent variable of executive function. This approach excludes measurement errors in our executive function factor, thus increasing its reliability and validity. However, the latent variable comprising executive function obtained only partial invariance which might have conceptual implications (Putnick and Bornstein, 2016). Yet, partial invariance across time for executive functions might be expected in this case. Executive functions in these fifth-grade children typically undergo developmental changes giving rise to changes in how such functions are conceptualized. Moreover, through learning and experience children might acquire skills that reorganize and expands their cognitive abilities which as well have implications for how executive functions are cognitively processed (Putnick and Bornstein, 2016). Furthermore, the analyses of several predictors, gives the opportunity to investigate different indices of physical activity to both executive function and academic performance. However, this also increases the chance of performing Type 1 errors, meaning that our results should be interpreted cautiously.

## Conclusion

The results from the present study revealed that executive function generally does not mediate the prospective relation between indices of physical activity and academic performance in 10-year-old Norwegian children over a period of 7 months. The modest mediation effect of executive function observed in the relation between motor skills and academic performance, as well as the direct link of the two, suggests that promoting physical activity that includes novel and complex motor tasks could be a useful approach for improving academic performance in children of this age group. Although, this finding should be interpreted carefully.

## Author contributions

KA conceived the idea for the paper together with YO, performed the data collection, analyzed the data, and wrote the manuscript draft. YO helped out in interpretation of the results and drafting the manuscript. EA contributed in data analyses and drafting the manuscript. KB composed the test battery concerning executive function. AL contributed in data analyses. GR obtained funding for the study. VM contributed in data collection and drafting the manuscript. All authors read, commented on, and approved the final manuscript.

### Conflict of interest statement

The authors declare that the research was conducted in the absence of any commercial or financial relationships that could be construed as a potential conflict of interest.
