# Supplementary material for: Executive Functions Do Not Mediate Prospective Relations between Indices of Physical Activity and Academic Performance: The Active Smarter Kids (ASK) Study
Source: Front Psychol. 2017 Jun 29;8:1088. doi: 10.3389/fpsyg.2017.01088 (PMC5489630; doi:10.3389/fpsyg.2017.01088)
Supplement: Supplementary file 1 [file DataSheet1.doc]

Assessed for eligibility (n= 105 [1395])

Excluded (n= 45 [193])

♦  Not meeting inclusion criteria (n= 44 [131] )

♦  Other reasons (n= 1 [62] )

Randomized (n= 60 [1202])

Schools declined to participate (n= 3 [27])

Children declined to participate (n= 0 [30])

Drop out (n= 0 [16])

♦  Withdrawing (n= 0 [5])

♦  Moved (n= 0 [8])

♦  Dropped out (n= 0 [3])

**Included in the analyses (n= 57 [1129])**

Missing data at follow-up:

♦ Accelerometry (n= 0 [107])

♦ Aerobic fitness (n= 0 [95])

♦ Shuttle Run (n= 0 [66])

♦ Aiming (n= 0 [51])

♦ Catching (n= 0 [52])

♦ Stroop CW (n= 0 [34])

♦ Verbal Fluency (n= 0 [32])

♦ WISC-IV backwards (n= 0 [34])

♦ TMT-b (n= 0 [64])

♦ Numeracy (n= 0 [83])

♦ Reading (n= 0 [113])

♦ English (n= 0 [79])

Lost to follow up (moved) (n= 0 [7])

Missing data at baseline:

♦ Accelerometry (n= 0 [124])

♦ Aerobic fitness (n= 0 [84])

♦ Shuttle Run (n= 0 [46])

♦ Aiming (n= 0 [36])

♦ Catching (n= 0 [96])

♦ Stroop CW (n= 0 [41])

♦ Verbal Fluency (n= 0 [34])

♦ WISC-IV backwards (n= 0 [36])

♦ TMT-b (n= 0 [78])

♦ Numeracy (n= 0 [49])

♦ Reading (n= 0 [63])

♦ English (n= 0 [67])

♦ Age (n= 0 [0])

♦ Sex (n= 0 [0])

♦ Socio economic status (n= 0 [60])

♦ Body fat (n= 0 [45])

♦ Pubertal stage (n= 0 [48])
